# From Crisis to Care: A Multidisciplinary Care Framework for Repatriated Civilian Hostages: Development, Implementation, and Early Outcomes

**DOI:** 10.3390/healthcare14183057

**Published:** 2026-09-17

**Authors:** Amir Nutman, Liron Yosha Orpaz, Shirly Oren, Yasmin Maor, Adam Lee Goldstein, Orit Twito, Doron Menachemi, David Hovel, Iris Zohar, Katia Dayan, Giulia Barda, Anat Levy, Zehavit Shpitzer, Liron Levinberg, Michal Noah, Orna Zvi, Anat Engel

**Affiliations:** 1 Hospital Management, Edith Wolfson Medical Center, Holon 5822012, Israel; 2 Faculty of Medical and Health Sciences, Tel-Aviv University, Tel-Aviv 6997801, Israel; 3 Department of Nephrology, Edith Wolfson Medical Center, Holon 5822012, Israel; 4 Rheumatology Unit, Edith Wolfson Medical Center, Holon 5822012, Israel; 5 Infectious Disease Unit, Edith Wolfson Medical Center, Holon 5822012, Israel; 6 Trauma Surgery Unit, Edith Wolfson Medical Center, Holon 5822012, Israel; 7 Endocrinology Unit, Edith Wolfson Medical Center, Holon 5822012, Israel; 8 Internal Medicine F and Department of Cardiology, Edith Wolfson Medical Center, Holon 5822012, Israel; 9 Internal Medicine E and Gastroenterology Unit, Edith Wolfson Medical Center, Holon 5822012, Israel; 10 Department of General Surgery B, Edith Wolfson Medical Center, Holon 5822012, Israel; 11 Department of Obstetrics and Gynecology and The Center for Care of Sexual Assault Victims, Edith Wolfson Medical Center, Holon 5822012, Israel; 12 Nutrition Department, Edith Wolfson Medical Center, Holon 5822012, Israel; 13 Social Services, Edith Wolfson Medical Center, Holon 5822012, Israel; 14 Office of Patient Experience, Edith Wolfson Medical Center, Holon 5822012, Israel; 15 Nursing Services and Quality Management, Edith Wolfson Medical Center, Holon 5822012, Israel

**Keywords:** repatriated civilian hostages, trauma-informed care, refeeding syndrome, captivity outcomes, hospital preparedness

## Abstract

**Objective**: On 7 October 2023, Hamas-led militants attacked Israeli communities bordering the Gaza Strip, abducting 251 people, including 201 civilians. Wolfson Medical Center was chosen by Israel’s Ministry of Health to care for repatriated civilians following a hostage release deal. We describe the development and implementation of a multidisciplinary care framework and report the clinical characteristics and short-term hospital outcomes of the repatriated civilians treated within it. **Methods**: A multidisciplinary framework was developed and implemented to address acute and chronic medical conditions, physical trauma, sexual assault, psychosocial support, and nutritional rehabilitation. Clinical characteristics and short-term hospital outcomes were retrospectively extracted from the electronic medical records. **Results**: Between 24 November and 1 December 2023, we treated 10 repatriated female hostages (median age 67 years, range 29–85 years) held captive for 48–55 days. They suffered physical trauma and loss of family members during the 7 October events. Captivity was associated with inadequate nutrition, poor hygiene, physical injuries, psychological distress, and insufficient management of chronic medical conditions. Acute conditions on arrival included rapid atrial fibrillation, heart failure, deep vein thrombosis, endocrine abnormalities, gastrointestinal symptoms and dermatologic conditions. Care required individualized planning, flexibility, and close coordination with national authorities, community health providers, and social services. **Conclusions**: Our experience highlights the need for comprehensive, coordinated care frameworks that not only address the medical, psychological, and social complexities of early recovery and reintegration but also ensure effective oversight and training of the medical teams involved.

## 1. Introduction

On 7 October 2023, at 6:29 AM, Hamas-led militants from the Gaza Strip infiltrated more than 20 Israeli communities, military bases, and an outdoor music festival. They subsequently committed widespread acts of violence and sexual assaults, murdered over 1200 people, and injured over 5500 [1,2]. During these attacks, Hamas and others kidnapped 251 people, including 201 civilians, into the Gaza Strip [3].

Taking hostages is not uncommon during military disputes; however, typically soldiers are those held in captivity. Thus, prisoners of war are generally healthy, fit, young men. However, on 7 October, the abductees were mostly civilians from a wide array of ages ranging from 9 months to 85 years, with various comorbidities [4].

Although abductions and prolonged captivity occur in diverse conflict settings, evidence specifically addressing the hospital-based care of repatriated civilian hostages remains limited. Relevant insights can nevertheless be drawn from studies of former prisoners of war and survivors of prolonged captivity, which document physical, functional, and psychological consequences and support coordinated medical, psychosocial, and rehabilitative care after release [5,6,7,8,9].

Edith Wolfson Medical Center (WMC) (Holon, Israel) was one of six hospitals selected by the Israeli Ministry of Health to treat the repatriated civilian hostages following a hostage release deal. Information regarding the medical status of the abductees in Gaza, and the conditions in which they were held in captivity, was sparse to nonexistent [4,10]. Therefore, we were challenged with preparing to manage a variety of medical conditions and severities [11].

We developed and implemented a comprehensive multidisciplinary framework comprising clinical protocols, trauma-informed care, and coordination with national response systems. The aim of this study was to describe the development and implementation of this framework and to report the clinical characteristics and short-term hospital outcomes of the repatriated civilians treated within it.

## 2. Methods

### 2.1. Study Design

This was a descriptive study of the development and implementation of a multidisciplinary care framework, combined with a retrospective review of the clinical characteristics and short-term hospital outcomes of the repatriated civilians treated within it.

### 2.2. Setting

WMC is an academic 726-bed hospital serving a population of approximately one million residents in central Israel. A dedicated “repatriation ward” was established to provide a private and secure setting for the initial hospital care of repatriated hostages.

### 2.3. Framework Development

A multidisciplinary “repatriation team” was assembled, consisting of specialists in internal medicine and relevant subspecialties, pediatrics, trauma surgery, gynecology and sexual assault victim care, nurses, social workers, and dietitians. The team reviewed available literature, identified likely clinical, psychological, nutritional, and social needs, and developed the care protocols through preparatory meetings and multidisciplinary expert consensus.

### 2.4. Simulation-Based Training and Drills

To enhance preparedness and refine the care protocols, the repatriation team conducted training and simulation exercises designed to address the complex challenges of treating repatriated hostages [12]. Actors portrayed scenarios, including the arrival of a medically unstable patient, a sexual assault victim, and the delivery of bad news, enabling healthcare professionals to practice critical skills in a controlled environment. These exercises helped identify gaps in the protocols, foster teamwork, and prepare staff for the anticipated clinical and emotional challenges.

### 2.5. Data Sources and Collection

Information on framework development and implementation was derived from the preparatory meeting records, care protocols, simulation materials, and operational documentation generated during the repatriation process.

Clinical and demographic data were retrospectively extracted from the electronic medical records of the repatriated civilians treated at WMC. Data extracted included age, duration and conditions of captivity, pre-existing medical conditions, captivity-related symptoms and injuries, acute conditions identified on arrival, weight loss, risk of refeeding syndrome, length of hospitalization, and discharge destination. Clinical data were extracted by study investigators who had also been members of the treating team. Members of the repatriation team were also invited to provide written reflections on the preparation and care process. Selected excerpts were included to illustrate the provider experience and practical lessons and were not subjected to formal qualitative analysis.

## 3. Results

### 3.1. Components of the Multidisciplinary Care Framework

The resulting framework addressed five principal domains of care: chronic and acute medical conditions, physical trauma and surgical care, sexual assault victim care, nutritional rehabilitation, and psychosocial support.

The detailed multidisciplinary care protocol is provided in Supplementary Section S1, and the nutritional rehabilitation protocol is provided in Supplementary Section S2.

#### 3.1.1. Chronic and Acute Medical Conditions

It was to be expected that chronic medications were not given (or only partially available) in captivity. Therefore, common chronic diseases may be exacerbated, and new medical conditions could emerge, including infectious diseases, venous thromboembolism, pressure ulcers, and physical deconditioning due to immobilization [4,13,14] (see Supplementary Section S1, Medical admission and assessment).

#### 3.1.2. Physical Trauma and Surgical Care

Physical injury may occur during events leading to the capture of the hostages, during the time of capture and ensuing transport, or during captivity. During captivity, a broad spectrum of injuries may occur, such as any means of torture/abuse, physical constraints, or surgical procedures performed on the hostages [6]. Therefore, preparation for acute traumatic injuries and post-acute sequelae such as infection, unset fractures, and complicated wounds was essential [15] (see Supplementary Section S1, Medical admission and assessment).

#### 3.1.3. Sexual Assault Victim Care

Sexual assault may occur during events leading to the capture of hostages or during captivity [2]. Thus, a gynecologist and a social worker, both specialized in sexual assault victim care, met each repatriate in their room, conducted a brief evaluation and privately offered to extend the conversation and examination. A forensic physician was on call to collect forensic evidence. Documentation in this context was intended to substantiate war crimes rather than to convict a specific perpetrator (see Supplementary Section S1, Addressing sexual assault and/or torture).

#### 3.1.4. Nutritional Rehabilitation

Proper nourishment is particularly crucial for the recovery phase after repatriation. The nutrition protocol was designed to address several issues, including prolonged stay in captivity, a wide range of ages and underlying illnesses, and the assumption that the food supply in captivity was poor and the repatriates’ caloric intake was very low.

A main concern was that the repatriates would arrive in a poor nutritional state and in danger of developing refeeding syndrome (RFS) [16,17]. Preventive measures included monitoring, initiating feeding gradually, and family education to avoid unregulated feeding upon arrival [18] (see Supplementary Section S1, sections on Nutritional support and Nutritional assessment, and Supplementary Section S2).

#### 3.1.5. Psychosocial Support

The population invaded and abducted on 7 October had been under the attack of rockets and mortars from Gaza for the previous 20 years, and therefore many suffered from long-term psychological and bio-psychological effects [19]. As such, the psychosocial intervention was tailored to this specific population. Another challenge was the lack of full knowledge that the repatriates had regarding the events of the 7 October massacre and the ensuing weeks, including devastating information pertaining to loved ones, community members, and their homes [1].

Planned interventions encompassed family preparation for the reunion with the repatriates, addressing various coping styles within the family, providing support for the repatriates’ immediate needs, and techniques for stress reduction and emotional support during challenging moments (see Supplementary Section S1, Emotional support).

Furthermore, depression, anxiety, and delirium were expected after prolonged captivity [7]. A psychologist and psychiatrist were on call for consultation as needed.

### 3.2. Framework Implementation and Stages of Care

**Figure 1:**
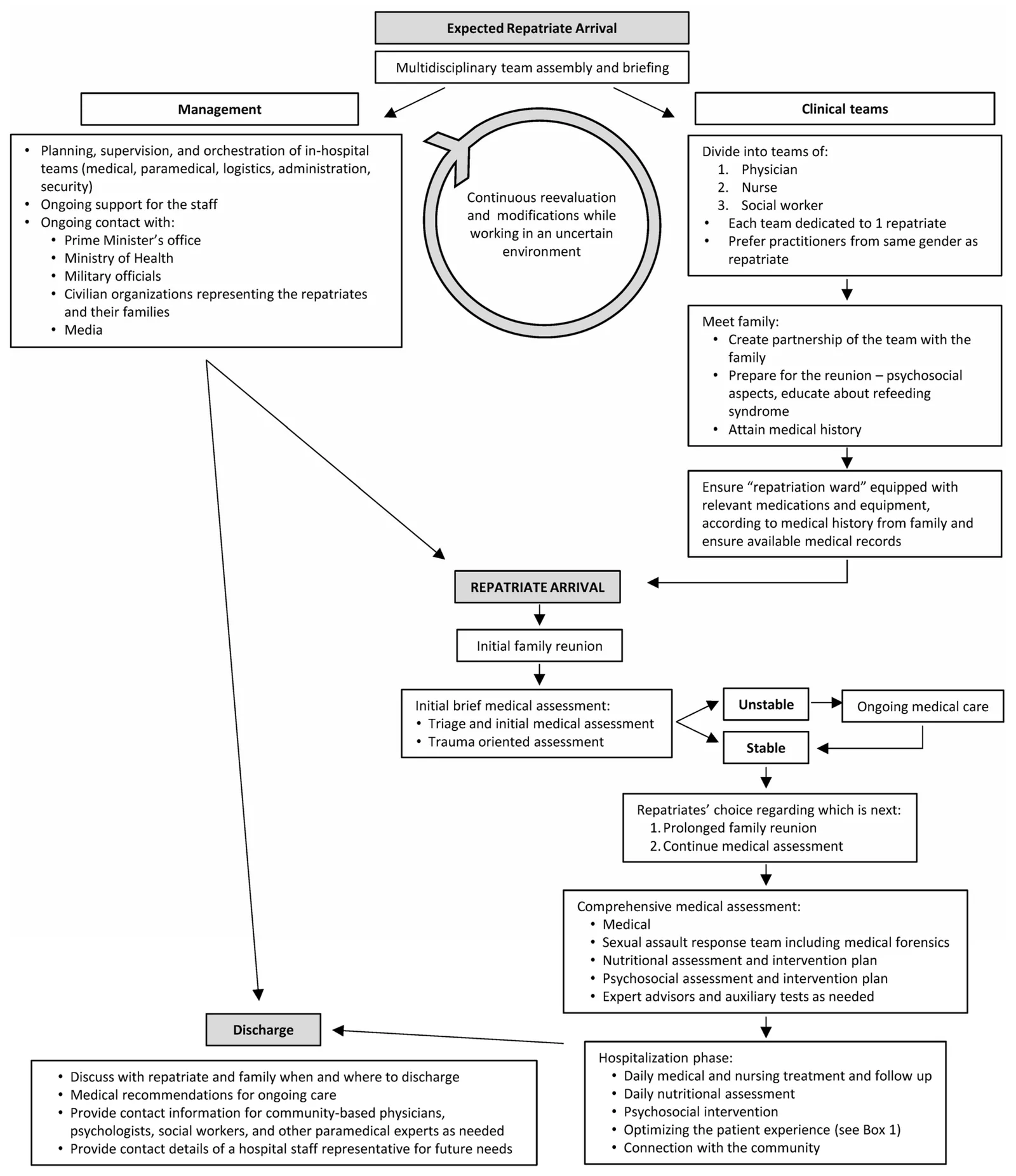
Treatment of repatriated hostages—schematic summary.

The framework was implemented in the care of 10 repatriated female civilian hostages treated at WMC between 24 November and 1 December 2023. Figure 1 summarizes the principal stages of care.

#### 3.2.1. Pre-Arrival Planning

When the identity of the hostages expected to be released was revealed, a triad consisting of a case-manager physician, a nurse, and a social worker, preferably of the same gender as the repatriate, were allocated as a dedicated team for each repatriate. The same team accompanied the family in preparation for the repatriate’s arrival and continued to attend the repatriate throughout the initial assessment and hospitalization stages. The concept of a “dedicated team” was established to create a safe and stable connection with the repatriate and their family, in medical and psychosocial aspects.

#### 3.2.2. Arrival and Initial Assessment

Upon arrival at the repatriation ward, the dedicated team performed initial triage, provided emergency care as needed, and prioritized treatment. A trauma surgeon examined each repatriate upon arrival, and a gynecologist was consulted for all patients, even if a full exam was not required. Additionally, nutritionists assessed the risk of RFS and provided tailored recommendations for initiating feeding. Specialists outside of the dedicated team were utilized to address specific medical concerns as needed.

#### 3.2.3. Hospitalization and Ongoing Care

Once the repatriates were considered stable, the treatment transitioned into the “hospitalization phase”, which focused on comprehensive treatment, emotional support, and patient education. Discharge decisions were based on the physical and mental health of each repatriate. Social workers worked closely with families and facilitated ongoing support from community resources after hospital discharge to aid in societal reintegration.

### 3.3. Clinical Characteristics and Short-Term Hospital Outcomes

To protect the privacy of this vulnerable population, clinical characteristics and hospital outcomes are reported only in aggregate. The duration of captivity was 48–55 days. The median age was 67 years (range 29–85 years), all were women whose residences were in communities bordering Gaza, and eight of them had suffered the loss of family members during the events of 7 October. During captivity, they reported being held with other hostages or alone, both above and below ground in tunnels, where the hygienic conditions were poor with limited access to toilets and showers. Food intake was inadequate, and access to medications was erratic.

Eight had prior chronic medical conditions which required medications. Seven sustained physical injuries during abduction or captivity, including shrapnel wounds, fractures, lacerations, and abrasions. Four reported diarrhea during captivity, and three had a febrile episode, of whom one was treated in a medical facility due to a wound infection.

All but one patient were medically stable upon arrival, although evidence of unmet medical needs was common. Cardiovascular conditions on arrival included poorly controlled hypertension, rapid atrial fibrillation complicated by acute heart failure, and deep vein thrombosis. Endocrine abnormalities included hyperglycemia and abnormal thyroid function. Gastrointestinal symptoms included diarrhea and constipation. Dermatologic findings included wounds at various stages of healing, fungal skin infections, and a pressure ulcer. Infectious diagnoses included rhinovirus infection associated with fever and gastrointestinal infection caused by nontoxigenic *Vibrio cholerae*. Sleep disturbances were reported in eight of the ten patients, while only a few required intervention for acute pain, anxiety, or mood symptoms. The median weight loss was 9.6% of body weight (range 0–25%) and five patients were assessed as being at high risk for RFS.

The median duration of hospitalization was 2 days (range 1–5 days). All patients had to be discharged to alternate places of residence as their homes were either destroyed or inaccessible. One patient was discharged to a subacute care facility.

### 3.4. Individualized, Trauma-Informed Care

Restoring the repatriates’ sense of control and self-esteem was a central component of care. This included restoring physical aids (such as walkers, hearing aids, glasses) and offering hygiene products and grooming services. In addition to immediate medical and psychosocial care, circumstance-specific and patient-specific supportive measures were implemented (Box 1). No outside media was allowed near the repatriated hostages’ point of arrival, and they entered the building through a back door away from the main entrance. A red carpet and additional lighting were installed at the improvised entrance to reduce any resemblance to the tunnels where they had been held captive. Furthermore, families (and pets when relevant) awaited the repatriated hostages in their private rooms upon their arrival. Privacy was maintained during the initial reunion between the repatriates and their loved ones, underscoring the importance of family preparation (see Supplementary Section S1, Before the arrival of the repatriate). Only once the patients and their families were settled and comfortable, a comprehensive evaluation began.

Box 1 **Unique situations addressed during treatment of repatriated civilian hostages.**

Basic concepts:

1. Ensuring a positive patient experience is crucial throughout the treatment process.
2. Each person has unique pillars of emotional strength and stability.
3. Self-esteem and self-perception may be harmed due to the abduction, confinement, emotional trauma, and inability to perform basic hygiene routines in captivity.

Goals:

1. Restore and maintain medical well-being.
2. Restore the basic needs that were deprived in captivity.
3. Recreate a sense of safety, normality, and freedom.
4. Provide personal and tailored care for the repatriates and families.
5. Recruit resources tailored to meet each repatriate’s specific needs.

Examples:

- Optometrist recruited for rapid adjustment of prescription eyeglasses.
- Audiologist recruited for replacement of lost hearing aids.
- Watches pre-ordered for repatriates to assist orientation.
- A bag filled with new clothes, personal hygiene products, and products for lice treatments was prepared for each repatriate, according to their specific measures and anticipated needs.
- After excluding risk for refeeding syndrome, a sensible amount of sweets and special meals were made available at the discretion of each repatriate.
- Grooming services were brought to the ward and each repatriate chose whether to utilize these services. This proved to be a meaningful service, since some of the hostages had returned from captivity with burnt and cut hair.
- Due to distorted circadian rhythms and insomnia, treatment times were adjusted to the patient rather than the staff.
- Repatriates’ dogs were accommodated in the department, as this was considered important for their well-being.
- One repatriate was traumatized from opening of the door in her room in captivity, and this feeling extended into the hospital stay. Therefore, a “do not enter” sign was posted on the door, and the family was instructed to contact the staff when the repatriate required ongoing care.
- The younger repatriates were eager to spend time outdoors with their family and friends; therefore, a secluded, secure, outdoor space was arranged for their convenience.
- After receiving consent from those involved, a sense of community and quality family time were emphasized by hosting a festive Shabbat (Friday night) dinner in the ward, during which extended family and community members were invited to join.

### 3.5. Reflections and Lessons from the Provider Experience

Written reflections from members of the repatriation team highlighted professional, organizational, and emotional aspects of preparation and providing care under exceptional circumstances. Selected reflections, including perceived strengths and areas for improvement, are presented in Box 2.

Box 2 **Insights from the practitioners in the “repatriation ward”.**

Aspects that were well addressed:

- The arrival of the hostages released from captivity is an exciting and extraordinary event for the hospital personnel. Therefore, it’s imperative to plan beforehand and work based on a written protocol.
- I felt we prepared thoroughly for the repatriates’ arrival—we planned ahead and early on, divided into teams, and considered many details regarding their arrival.
- The teams were precisely chosen based on professional expertise, as well as personality traits and character, and were a good fit for the different needs of the repatriates.
- Having a large, professional, multidisciplinary team created the feeling that we can handle any medical challenge that may appear, from the most trivial to life-threatening situations.
- We nervously anticipated the abductees’ return alongside their families for hours, from the initial photos of their transfer to the Red Cross until they finally arrived at our hospital. This tormenting experience gave me a better perspective of the torture the families were enduring for many weeks in anticipation of their loved ones’ return, generating even more empathy and compassion towards them during this initial connection.
- After the first night in the hospital, the young repatriates looked healthy and physically ready to leave. At this point, I felt our goal was not medical per se, but to create a safe space for them in the hospital, shielding them from the reality that awaits outside.

Thoughts for improvement:

- Information regarding the medical state of the repatriates was delivered only a short time before their arrival at the hospital. We should strive to pass on medical information as soon as possible to allow appropriate adjustments.
- We should be very mindful of the importance of protecting the repatriates’ privacy, especially during the initial meeting with their families.
- I believe the original care team should be tasked with the responsibility of closely following up with each repatriate through weekly calls after discharge, to monitor reintegration, address emerging medical and social needs, and provide support using hospital resources.
- We should encourage the repatriates to stay in the hospital for as long as their physical and emotional conditions require.
- I felt the hostages who had returned needed a longer adjustment phase before returning to their everyday life. Perhaps we should offer them a transitional care facility, where they can lengthen their stay between discharge from the hospital and return to the community.
- Collaboration with respective repatriation teams in other hospitals during preparation and treatment could positively impact preparedness for different situations and coping of the staff with this unprecedented challenge.

Thoughts and feelings regarding this unique experience:

- The initial appearance and apparently good physical condition of the women who returned from captivity does not at all indicate the physical and mental suffering they went through.
- Connecting with the repatriate’s family, especially before her arrival, was difficult. I felt the family members were guarded and difficult to appease, as they felt betrayed by the country and its authorities.
- I felt the hospital made an extraordinarily thoughtful effort for a patient-centered approach. The repatriates and families were appreciative of this, however all the materialistic efforts seemed superficial and disproportionate to the deeply rooted psychological stress with which they were dealing. I still feel powerless regarding the horrendous experience they endured.

## 4. Discussion

We developed a structured framework for the acute hospital care of repatriated civilian hostages abducted from Israel on 7 October 2023 and held in captivity in Gaza. The physical and psychological condition of the hostages before arrival was largely unknown, requiring a flexible and multidisciplinary approach. The framework was therefore designed to accommodate wide variation in age, sex, injuries of different types and severity, new or worsening medical conditions, possible sexual assault, and psychological distress. Trauma-informed care was particularly relevant because captivity involved profound loss of control, uncertainty, and repeated exposure to threats. Accordingly, the framework emphasized safety, privacy, choice, restoration of autonomy, and avoidance of potentially retraumatizing interactions [20,21].

To effectively manage these complex cases, a multidisciplinary team was assembled. This is consistent with hospital disaster-management literature emphasizing integrated, multidisciplinary preparedness and response [9,22]. Simulation-based training, including drills with actors, was conducted to prepare for different scenarios. This approach has been shown to strengthen team preparedness, multidisciplinary coordination, and clinical decision-making in complex emergency settings [12,22,23]. Given the emotional burden on healthcare workers and the potential for compassion fatigue and secondary traumatic stress, ongoing emotional support was made available to the medical teams [24,25].

Comparable approaches have been described in other hostage and repatriation settings. The International Committee of the Red Cross has proposed a seven-phase care model for humanitarian workers taken hostage, incorporating a multidisciplinary crisis-management structure that includes security, human resources, communications, and clinical teams [26]. This model shares with our framework an emphasis on multidisciplinary coordination, but differs in its greater focus on long-term follow-up, peer debriefing, and professional reintegration, reflecting differences in the characteristics and anticipated needs of the respective hostage populations.

Hospital management played an important role in resource allocation, staff selection, and protocol validation. Coordination between healthcare providers, government agencies, and community organizations supported both acute care and transition planning [22]. This collaboration included arrangements for continuity of care beyond hospitalization through community-based medical and social services.

Recovery after prolonged captivity extends beyond acute stabilization and may require rehabilitation, psychological support, continuity of care, and assistance with community reintegration [9,27]. Although the present report focuses on the acute hospitalization phase, discharge planning needed to account for the combined effects of disrupted management of chronic conditions, physical and emotional trauma, nutritional deprivation, loss of autonomy, and displacement from home and community.

Several protocols developed in Israel following the 7 October attacks have addressed complementary aspects of hostage repatriation, including initial military medical contact [28], care of children and family groups [29], and the care of repatriated hostages of foreign citizenship and diverse cultural backgrounds [30]. These reports highlight the need to tailor repatriation care to patient age, family structure, cultural context, and the setting of first medical contact. The framework described here was developed for adult civilian hostages and was implemented in repatriated women across a wide age range.

Taken together, these approaches suggest that certain principles, particularly multidisciplinary coordination, trauma-informed care, and continuity planning, may be broadly applicable across hostage repatriation settings. However, specific organizational structures and implementation strategies are likely to remain context-dependent and should be adapted to the characteristics of the repatriated population and the local healthcare and organizational context.

## 5. Limitations

The framework was developed in the context of limited prior literature and therefore relied substantially on expert opinion to anticipate potential clinical scenarios. It was also tailored to the specific circumstances of the 7 October attack, including the demographic characteristics of the hostages, captivity conditions, and duration of detention. The framework was implemented at a single hospital and applied to only 10 repatriated civilians, limiting assessment of its performance in larger or more diverse populations and in other healthcare settings. Future adaptations should account for variations in captivity conditions, hostages’ medical and psychological profiles, healthcare systems and geopolitical contexts. In addition, most reported outcomes were based on clinical observations rather than standardized or validated outcome measures. Follow-up was limited to the hospitalization period, yet the challenges faced by repatriated individuals extend well beyond discharge. Longer-term monitoring is needed to assess the consequences of captivity and further refine treatment and support frameworks.

## 6. Conclusions

We present the development, implementation, and early outcomes of a structured, coordinated care framework for treating repatriated civilian hostages and suggest that a trauma-informed, multidisciplinary approach may support early patient recovery and reintegration. Although developed for the specific circumstances of the 7 October hostage release, elements of the framework may be applicable to other situations involving the repatriation of civilians after prolonged captivity, provided that they are adapted to the local clinical, organizational, and geopolitical contexts.

## Data Availability

The data that support the findings of this study are not publicly available due to the sensitive nature of the subject matter.

## Supplementary Materials

The following supporting information can be downloaded at: https://www.mdpi.com/article/10.3390/healthcare14183057/s1, Section S1: Wolfson Medical Center protocol for medical treatment of repatriated hostages; Section S2: Guidelines for risk assessment and prevention of refeeding syndrome among adult repatriates. (see Refs. [18,31]).

## Abbreviations

WMC—Wolfson Medical Center; RFS—refeeding syndrome.
