# Supplementary material for: From Crisis to Care: A Multidisciplinary Care Framework for Repatriated Civilian Hostages: Development, Implementation, and Early Outcomes
Source: Healthcare (Basel). 2026 Sep 17;14(18):3057. doi: 10.3390/healthcare14183057 (PMC13606442; doi:10.3390/healthcare14183057)
Supplement: Supplementary file 1 [file healthcare-14-03057-s001.zip › healthcare-4444730-supplementary.pdf]

From crisis to care: a multidisciplinary care framework for repatriated civilian hostages

- development, implementation, and early outcomes

Authors: Amir Nutman*, Liron Yosha Orpaz*, Shirly Oren*, Yasmin Maor, Adam Lee Goldstein, Orit Twito, Doron Menachemi, David Hovel, Iris Zohar, Katia Dayan, Giulia Barda, Anat Levy, Zehavit Shpitzer, Liron Levinberg, Michal Noah, Orna Zvi, and Anat Engel

*Authors with equal contribution

Supplemental Material – Contents

Section S1: Wolfson Medical Center protocol for medical treatment of repatriated hostages

Section S2: Guidelines for risk assessment and prevention of refeeding syndrome among adult repatriates

Section S1: Wolfson Medical Center protocol for medical treatment of repatriated hostages

As formulated for the return of abductees from Gaza during the Israel-Hamas ("Swords of Iron") war in 2023.

Before the arrival of the repatriate (from now on regarded as "patient" for simplicity)

A team consisting of three members – a physician case manager, nurse, and social worker – will be assigned to each specific patient.

The team will meet the patient's family as they arrive to the hospital, for the following purposes:

- Introduce the team members and their roles.
- Explain about the planned scheme of events upon their loved one's arrival.
- Get personally acquainted with the family, to make them feel comfortable with the team members and gain their trust. It is important to approach the family as collaborators in the mutual mission of making the patient feel comfortable and safe.
- Understand the basic family pedigree, and which family members were also assaulted, *i.e.* injured and to what extent, killed, abducted, or missing.
- Gather information concerning the patient's past medical history, chronic medical conditions (that may have exacerbated in captivity), chronic medications, and use of special aids (glasses, hearing aids, dentures, etc.).
- Receive information about the patient's state in captivity, from official authorities or hearsay, to help the team and the family together to assume the patient's possible physical, psychological, sociological, and spiritual needs.
- Prepare the family for possible physical consequences of captivity.
- Prepare the family for psychosocial aspects of the reunion, and discuss various coping styles relevant to the specific family and patient.
- Educate the family regarding refeeding syndrome and dietary precautions (see below - Nutritional assessment).

Upon arrival of the patient

- The family will meet with the patient as they enter the dedicated ward in the hospital.
- The medical team will meet with the patient as they enter their private room with the family, introduce themselves, and assess (triage) the patient's physical status by observation and short conversation.
- If the patient is considered to be in stable condition, the staff will ask the patient and their family to choose if to complete the medical assessment immediately, or to give them private time together and return later.
- If the patient requests time with the family, the family will be asked to call upon the team when ready.

Nursing admission and assessment

- Document vital signs: heart rate, blood pressure, temperature, oxygen saturation, and pain level.
- Document physical parameters: height, weight, body mass index.
- Obtain laboratory tests, as detailed below.

Medical admission and assessment

Conducted by an internal medicine expert, preferably from the same gender as the returning hostage.

<u>Medical history</u>:

- As per any patient admission, including past medical history, chronic medications, allergies
- A brief account of the *medically relevant* events prior to the abduction, during the abduction, and during captivity, including medical and surgical treatments. An emotional assessment will be formulated by the social worker with expertise in traumatic situations.

- Full review of systems, as well as questions oriented to complications of captivity:
  - General health and basic nutritional assessment – What were the height and weight before abduction? What did they eat during captivity, how much?
  - Eyes – Regular use of eyeglasses? Injuries? Are there pain, secretions, or blurry vision?
  - Ears – Regular use of hearing aids? Injuries? Are there pain, secretions, or hearing loss?
  - Mouth and teeth – Regular use of dentures? Injuries, broken teeth? Are there pain, swelling, ulcers?
  - Skeletal system – Injuries and wounds? Were injuries treated in captivity? If so, in what way and when? Is there currently pain or a limitation of motion in the neck, back, or limbs?
  - Cardiorespiratory – Injury to the chest? Pain in the chest, palpitations, dyspnea, cough, or hemoptysis?
  - Gastrointestinal – Suffer from diarrhea, constipation, vomiting, abdominal pain, melena, or rectal bleeding – during captivity, or now?
  - Urine – Pain, burning sensation, frequency, hematuria?
  - Skin – Injuries such as scrapes, ulcers, rashes, burns, penetrating wounds, pruritus, or infection? Were they treated – when, how? Any current symptoms?
  - Pain anywhere?
- Physical examination: a full physical examination after undressing in the presence of one family member, per patient's request, with special emphasis on possible captivity-associated complications:
  - General condition, nutritional condition
  - Eye and ear, nose, throat (ENT) assessment, including teeth
  - Cardiorespiratory exam
  - Abdominal exam
  - Limbs – Search for wounds, fractures, dislocations, range of motion in all joints
  - Skin – Search for wounds, penetrations of foreign objects (such as bullets or shrapnel), rashes, infections, scabies, pediculosis
  - Neurological exam
- Laboratory tests:
  - Blood - Complete blood count, complete biochemical panel consisting of glucose, kidney function tests, albumin and liver functions tests, electrolytes and basic minerals (Na, K, Cl, Ca, P, Mg), CPK, iron panel (iron, transferrin saturation, ferritin), folic acid, vitamin B12, thyroid panel.
  - If suspected infection – C-reactive protein, cultures as appropriate.
  - If injuries or possible need for surgery – Clotting tests, blood type and crossmatch
  - Infectious serology - HIV, HBV, HCV, syphilis, urine PCR panel for sexually transmitted infections[1] – taken for assessment of infectious diseases contracted *via* blood or other body fluids. The patient should consent to these tests.
  - Females aged 12-50 – beta-HCG - the patient should consent to this test.
  - Urine for toxicology screen, and a sample refrigerated in the lab for further testing as needed - the patient should consent to this test.
  - If diarrhea – fecal samples for viral, bacterial, and parasitic pathogens.
  - Ask to reserve serum for additional tests as needed.
- Auxiliary diagnostic tests:
  - ECG – From the age of 40 years old, or if clinically needed
  - Chest X-ray – As needed
  - Additional radiological exams as necessary
- Call for expert advisors as necessary according to findings – traumatologist surgeons, gynecologist, ophthalmologist, orthopedic surgeon, otolaryngologist, infectious diseases specialist, etc.

Addressing sexual assault and/ or torture

The staff is advised not to ask the patient directly. Instead, let the patient know that the staff is here to assist in every way needed, and that physical examination by any relevant expert is available. Additional tests and emotional support are granted as per patient request. If a staff member is informed of assault, or suspects it, an expert in the field will be called upon.

In addition, a female gynecologist with expertise in sexual assault victim care will introduce herself to all the female patients and offer her help in whichever matter needed. The patient may choose to speak with the expert later on in the hospitalization.

Pain management

Pain will be treated with medication as per hospital protocol, according to a scale from mild to severe.

Nutritional support and vitamin supplementation

All patients are assumed to have vitamin deficiencies, due to impaired sun exposure and malnutrition. Therefore, all patients should be treated with vitamin supplements, as follows:

- Vitamin B1 (thiamine) – Every patient should receive one intravenous dose (100 mg thiamine in 100 ml normal saline) upon arrival. Patients with moderate or high risk for refeeding syndrome (see Section S2) receive 100 mg daily for 5 days. For children, the dose is 2 mg/kg (max. 100 mg/day).
- Vitamin D3 – Every patient should receive a single dose of 100,000 IU vitamin D3 solution upon arrival. For children, consult with a dosing table.
- A multivitamin – Every patient should receive one tablet once daily. For children, according to age.
- Iron, folic acid, vitamin B12 – As needed according to the blood work.

Nutritional assessment and dietary recommendations (see Section S2)

It is assumed that all the patients will have malnutrition of some degree. An increase in dietary intake following a significant period of impaired intake may cause **refeeding syndrome**[2]. This complication may occur within 72 hours in patients suffering from severe malnutrition including continuous starvation. The syndrome results from abruptly increased intake of carbohydrates.

To reduce the risk of refeeding syndrome:

- Upon the patient's arrival, the patient will be asked what she/he has eaten during the journey from captivity to the hospital.
- If the patient has not eaten yet, the patient may drink a drink sweetened with one teaspoon of sugar or eat a small treat (such as 3 plain cookies, or apple sauce without additional sugar). Representatives of state and families that met with the repatriates before arrival to the hospital should be informed of this recommendation in advance.
- The dietician will assess each patient immediately following the medical assessment. The dietician will deduce the risk of refeeding syndrome, and consequently advise the staff, patient, and family on further dietary recommendations, specifically recommended pace, amount, and dietary composition.
- Results of the electrolytes and minerals must be addressed before commencing feeding.
- Intravenous/ intramuscular thiamine will be given before commencing feeding, or within 30 minutes.
- If high risk for refeeding syndrome, during the first three days of feeding:
    1. Levels of phosphorus, potassium, and magnesium will be monitored and supplements given accordingly.
    2. A daily follow-up of the patient's fluid status, including peripheral edema, will be conducted.
- If low risk for refeeding syndrome, the patient may eat a normal diet, however instructed to avoid over-eating, especially of carbohydrates.
- The dietician will revisit the patient daily or as needed according to risk stratification.

Emotional support

If the team suspects that the patient suffers from emotional distress and may value additional support:

- Convey this to the social worker.
- A psychologist and/or psychiatrist will be called upon as needed.
- Aim for minimal use of anxiolytic medications, especially benzodiazepines, in order to facilitate healthy coping with this difficult situation and reduce substance dependence.

Decision to discharge from the hospital

The decision to discharge from the hospital will be based on the physical condition, as well as the emotional state of the patient, the readiness to meet the "outside world", as well as access to suitable living arrangements after discharge.

Upon discharge, the patient will receive:

- A discharge letter.
- Specified medical and dietary recommendations.
- A recommendation to continue a multi-vitamin tablet once daily for one month.
- A recommendation to mind oral hygiene and follow-up with a dentist.
- A recommendation for gynecologist follow-up (for women).
- A recommendation for professional emotional support, along with the name and contact details of a specified social worker from the community.
- A recommendation for continued care with a family physician, along with the name and contact details of a specified doctor who will be transferred the discharge papers.
- Contact details of a hospital staff representative that will be of service as needed.

Section S2: Guidelines for risk assessment and prevention of refeeding syndrome among adult repatriates

Reproduced with permission from recommendations constructed by the Israel Ministry of Health and conveyed to all hospitals receiving repatriates.

The following tables are general measures to be implemented along with the guidelines stated above in eText 1, and ongoing assessments of an experienced dietician.

Risk stratification for refeeding syndrome:

**Criteria**	**Moderate Risk (meets 2 criteria)**	**High Risk (meets 1 criterion)**
BMI (kg/m^2)	16-18.5	Less than 16
Weight loss	5% within a month, or 10-15% during the captivity period	More than 15% during the captivity period
Dietary intake	Reduced or none at all in the last 5-6 days<br>OR<br>50% - 75% of the energetic needs for 7 or more days during an acute medical state or trauma<br>OR<br>50% - 75% of the energetic needs for more than a month	Reduced or none at all for 7 or more days<br>OR<br>Less than 50% of the energetic needs for 7 or more days during an acute medical state or trauma<br>OR<br>Less than 50% of the energetic needs for more than a month
Plasma electrolyte concentrations – potassium, phosphorus, magnesium, calcium, and iron	Concentrations slightly low before beginning feeding<br>OR<br>Normal concentrations after a single dose of supplementation	Low concentrations before beginning feeding<br>OR<br>Normal concentrations after several doses of supplementation
Loss of muscle mass	Mild to moderate loss	Severe loss
Comorbidities	Present	Present

Feeding plan according to risk stratification:

**Target**	**To achieve full dietary needs within 5-10 days**	
**Micronutrient supplementation**	Oral/enteral feeding<br>Multivitamin supplement once daily for 10 or more days, according to individual dietary assessment<br>Vitamin B12, vitamin D, and iron as per medical recommendation	
**Fluid**	**Moderate Risk**	**High Risk**
	30-35 mL/kg	Begin 20-25 mL/kg for 3 days<br>Then 25-30 mL/kg for days 4-6<br>Then 30-35 mL/kg from day 7
**Enteral Feeding – Caloric Intake** (considering mainly carbohydrate calories)	**Moderate Risk**	**High Risk**
	10-15 kCal/kg during the first 24 hours	5-10 kCal/kg during the first 24 hours
	May begin IV glucose 100-150 grams during the first 24 hours<br>Must consider the caloric intake from IV dextrose-containing solutions and medications containing dextrose	
**Rate**	Gain 33% of the target every 1-2 days, with close monitoring of the caloric intake from carbohydrates	

References:

1. Workowski KA, Bachmann LH, Chan PA, et al. Sexually Transmitted Infections Treatment Guidelines, 2021. *MMWR Recommendations and Reports* 2021;70(4). doi:10.15585/mmwr.rr7004a1

2. da Silva JSV, Seres DS, Sabino K, et al. ASPEN Consensus Recommendations for Refeeding Syndrome. *Nutrition in Clinical Practice*. Published online 2020. doi:10.1002/ncp.10474
